# Comparative Immunoprofiling in Psoriasis and Psoriasiform Dermatitis Using Inflammatory and Structural Biomarkers: A Preliminary Study

**DOI:** 10.3390/diagnostics16183047

**Published:** 2026-09-20

**Authors:** Mihaela Paula Toader, Oana Mihaela Condurache Hritcu, Mirela Grigorovici, Stefan Toader, Carmen Solcan, Daciana Elena Branisteanu

**Affiliations:** 1 Department of Surgicals, Faculty of Dental Medicine, Grigore T. Popa University of Medicine and Pharmacy, 700115 Iasi, Romania; toaderpaula@gmail.com (M.P.T.); toaderstefan@gmail.com (S.T.); debranisteanu@yahoo.com (D.E.B.); 2 Pathology Laboratory, University Clinical Railways Hospital, 700506 Iasi, Romania; mirelagri@yahoo.com; 3 Department of Molecular Biology, Histology and Embryology, University of Life Sciences Ion Ionescu de la Brad, 700489 Iasi, Romania; carmensolcan@yahoo.com

**Keywords:** psoriasis, psoriasiform dermatitis, immunohistochemistry, ICAM-1, VCAM-1, IL-1β, IL-17A, TNF-α, diagnostic biomarkers

## Abstract

**Background:** Differentiating psoriasis from psoriasiform dermatitis can be challenging due to overlapping clinical and morphological features, especially in certain areas, such as the palms, soles and scalp. While the distinct roles of the Th17 and Th2 axes in these inflammatory dermatoses are recognized, the comprehensive in situ expression of adhesion molecules, inflammatory cytokines, and structural markers within a unified diagnostic framework remains incompletely characterized. **Objectives:** The aim of this study was to compare the immunohistochemical expression of intercellular adhesion molecule-1 (ICAM-1), vascular cell adhesion molecule-1 (VCAM-1), tumor necrosis factor-alpha (TNF-α), interleukin-1 beta (IL-1β), interleukin-17A (IL-17A), Collagen IV, and S100B in psoriasis and psoriasiform dermatitis and to evaluate their diagnostic and pathogenetic significance. **Methods:** This retrospective observational study evaluated skin biopsy specimens from 22 patients: 11 with psoriasis and 11 with psoriasiform dermatitis. Immunohistochemical staining was performed for seven markers. Expression intensity was semi-quantitatively scored (0–3) across four compartments: membranous, cytoplasmic, nuclear, and inflammatory infiltrate. Statistical analysis included Mann–Whitney U tests with effect size calculations and receiver operating characteristic (ROC) analysis with bootstrap confidence intervals. **Results:** Psoriasis lesions demonstrated significantly higher total expression scores compared with psoriasiform dermatitis for IL-17A (median 8 vs. 4, *p* < 0.001, *r* = 0.847), IL-1β (8 vs. 4, *p* < 0.001, *r* = 0.847), TNF-α (8 vs. 4, *p* < 0.001, *r* = 0.819), and ICAM-1 (9 vs. 4, *p* = 0.003, *r* = 0.637). VCAM-1 showed a higher median expression score in psoriasis (7 vs. 6), but the difference was not statistically significant (*p* = 0.134, *r* = 0.322). Structural markers Collagen IV (*p* = 0.110) and S100B (*p* = 0.504) showed no significant differences. ROC analysis yielded an AUC of 1.000 for both IL-17A and IL-1β, followed by TNF-α (AUC = 0.983) and ICAM-1 (AUC = 0.876). VCAM-1 exhibited moderate discriminative ability (AUC = 0.694). A combined biomarker panel (IL-17A, TNF-α, and ICAM-1) achieved an AUC of 1.000 within this study cohort; however, this exploratory finding requires external validation. **Conclusions:** IL-17A, IL-1β, TNF-α, and ICAM-1 represent the most discriminative immunohistochemical markers separating psoriasis from psoriasiform dermatitis. In this preliminary study, IL-17A, IL-1β, TNF-α, and ICAM-1 showed the greatest between-group differences and the highest diagnostic performance within the investigated cohort. VCAM-1 may provide complementary diagnostic information. Collagen IV and S100B appear less useful as standalone discriminators. These findings support the potential role of biomarker-guided precision dermatopathology, although validation in larger independent cohorts is required before clinical implementation.

## 1. Introduction

Inflammatory skin diseases represent a major global health burden, affecting hundreds of millions of individuals and substantially diminishing their quality of life through chronic symptoms, psychosocial stigmatization, and systemic comorbidities [1]. Among the most common histological patterns encountered in dermatopathology is the psoriasiform pattern, characterized by regular or irregular epidermal hyperplasia with elongation of rete ridges. This morphological pattern is the hallmark of psoriasis vulgaris, a chronic immune-mediated disease affecting approximately 2–3% of the global population, driven primarily by the Interleukin (IL)-23/T helper (Th) 17 axis [2]. However, a highly similar histological pattern—termed psoriasiform dermatitis—frequently occurs in the chronic stages of other inflammatory conditions, most notably atopic dermatitis (AD), allergic contact dermatitis, nummular eczema, and seborrheic dermatitis [3,4].

Although psoriasis and eczematous dermatoses are traditionally regarded as distinct entities, overlapping clinical, histopathological, and immunological features are increasingly recognized. Clinically, atopic dermatitis may present with psoriasiform lesions, while psoriasis can resemble eczema, making histopathological assessment important for differential diagnosis [5,6,7,8]. On the other hand, when lesions are limited to the scalp region, differentiating psoriasis from seborrheic dermatitis remains challenging due to overlapping clinical and histopathological findings [9].

The clinical and histopathological differentiation between psoriasis and psoriasiform dermatitis is clinically important, as accurate diagnosis informs therapeutic selection and prognostic counseling. Psoriasis vulgaris presents with well-demarcated, erythematous plaques covered by silvery-white scales, predominantly affecting extensor surfaces, the scalp, and the lumbosacral region. In contrast, the clinical presentation of psoriasiform dermatitis depends on the underlying condition and may include poorly defined erythematous, scaly or eczematous lesions, with distribution varying according to the specific diagnosis [4]. However, overlapping phenotypes are increasingly recognized, particularly in chronic lesions, pediatric populations, and certain ethnic groups where the classical morphological distinctions become blurred [5,6,7,8].

The advent of highly targeted biologic therapies has revolutionized dermatological management but simultaneously heightened the need for precise diagnostic endotyping. Targeted therapies directed against the IL-17 and IL-23 pathways have transformed the management of psoriasis, whereas blockade of IL-4/IL-13 signaling has become central to the treatment of AD [2,4]. Misdiagnosis can lead to therapeutic failure, unnecessary adverse effects, or paradoxical reactions—a phenomenon where biologic therapy targeting one disease axis induces the phenotype of the other [7]. These paradoxical reactions, such as dupilumab-induced psoriasiform eruptions or anti-TNF-induced eczematous dermatitis, underscore the delicate immunological balance between the Th17 and Th2 axes and highlight the clinical consequences of diagnostic imprecision [7].

In challenging scenarios where standard hematoxylin and eosin (H&E) staining reveals overlapping features—such as acanthosis with variable spongiosis, mixed parakeratosis and orthokeratosis, or atypical inflammatory infiltrates—definitive diagnosis may be challenging on morphological grounds alone [6]. Consequently, there is a critical need for reliable tissue biomarkers that can differentiate these conditions at the molecular level and provide objective diagnostic criteria.

The pathogenesis of psoriasis involves a complex interplay between keratinocytes, dendritic cells, T lymphocytes, and the vascular endothelium, orchestrated by a specific cytokine milieu [10,11,12,13,14,15,16]. Key inflammatory cytokines, particularly tumor necrosis factor-alpha (TNF-α), interleukin-1 beta (IL-1β), and interleukin-17A (IL-17A), act as central amplifiers of the psoriatic inflammatory cascade [17]. TNF-α and IL-1β activate the innate immune response and promote dendritic cell maturation, while IL-17A directly drives keratinocyte hyperproliferation and neutrophil recruitment [18]. Furthermore, leukocyte recruitment to the inflamed skin is tightly regulated by cellular adhesion molecules expressed on both keratinocytes and the vascular endothelium. Intercellular adhesion molecule-1 (ICAM-1/CD54) and vascular cell adhesion molecule-1 (VCAM-1/CD106) are crucial for the tethering, firm adhesion, and transendothelial migration of lymphocytes and monocytes [19,20,21]. ICAM-1 interacts with lymphocyte function-associated antigen-1 (LFA-1) on T cells, while VCAM-1 binds very late antigen-4 (VLA-4) on mononuclear cells, each mediating distinct aspects of the inflammatory cell trafficking cascade [22,23].

While the roles of these individual markers have been studied in various dermatoses, comprehensive comparative studies simultaneously evaluating their spatial distribution and diagnostic utility in differentiating psoriasis from psoriasiform dermatitis remain scarce. Previous immunohistochemical studies have typically focused on single markers or limited panels, and few have incorporated modern diagnostic performance metrics such as ROC analysis [24,25,26]. Furthermore, the combined evaluation of structural markers (Collagen IV for basement membrane integrity) and cellular markers (S100B) alongside inflammatory markers within a unified framework remains insufficiently explored.

This study provides a comparative immunohistochemical analysis simultaneously evaluating cellular adhesion molecules (ICAM-1, VCAM-1), key inflammatory cytokines (TNF-α, IL-1β, IL-17A), and structural/cellular markers (Collagen IV, S100B) in psoriasis and psoriasiform dermatitis within a unified diagnostic framework. The aim of this study was to compare the immunohistochemical expression of these seven markers in psoriasis and psoriasiform dermatitis, evaluate their diagnostic performance through ROC analysis, and assess their pathogenetic significance for differential diagnosis and immune profiling.

## 2. Materials and Methods

### 2.1. Study Design and Specimen Collection

This retrospective observational comparative study was performed using archived formalin-fixed, paraffin-embedded (FFPE) skin biopsy specimens obtained from the Department of Pathology, CF Clinical Hospital, Iași, Romania. Biopsy specimens collected between January 2015 and January 2023 were retrospectively reviewed.

The study included 22 patients, comprising 11 cases of clinically and histopathologically confirmed psoriasis vulgaris and 11 cases included in the heterogeneous psoriasiform dermatitis comparator group, consisting of five cases of atopic eczema, three cases of seborrheic dermatitis, and three cases of palmoplantar eczema, including one hyperkeratotic palmoplantar eczema (Table 1). All hematoxylin and eosin (H&E)-stained slides were independently reviewed by two experienced dermatopathologists to confirm the diagnosis before immunohistochemical analysis.

Inclusion criteria comprised: (1) age ≥18 years; (2) clinicopathological diagnosis of psoriasis vulgaris or one of the inflammatory dermatoses included in the psoriasiform dermatitis comparator group; (3) availability of well-preserved FFPE tissue suitable for immunohistochemical analysis; and (4) complete histopathological documentation.

Exclusion criteria included: (1) inadequate tissue preservation or fixation artifacts; (2) insufficient tissue for the complete immunohistochemical panel; and (3) incomplete histopathological documentation.

All tissue specimens were anonymized before analysis and evaluated exclusively for research purposes. The study was conducted in accordance with the Declaration of Helsinki and approved by the Ethics Committee of University Clinical Railways Hospital, Iasi, Romania (Approval no. 12449, Date of approval 14 July 2026).

### 2.2. Diagnostic Criteria and Clinical Data

For the individual diagnoses within the comparator group, atopic eczema was diagnosed according to the Hanifin and Rajka clinical criteria in conjunction with compatible histopathological findings. Palmoplantar eczema, including the hyperkeratotic variant, was diagnosed based on the characteristic palmoplantar clinical presentation and compatible histopathological findings, with particular attention to the exclusion of classical psoriasis. Seborrheic dermatitis was diagnosed based on its characteristic clinical morphology and distribution in seborrheic areas, together with compatible histopathological findings and the absence of a histopathological constellation diagnostic of classical psoriasis.

Histopathological distinction between classical psoriasis and the psoriasiform dermatitis comparator group was based on the overall pattern and constellation of microscopic features rather than on a single criterion. Classical psoriasis was characterized by regular psoriasiform epidermal hyperplasia with relatively uniform elongation of the rete ridges, confluent parakeratosis, thinning or focal loss of the granular layer, suprapapillary thinning, and elongated dermal papillae containing dilated and tortuous capillaries. Neutrophilic accumulation within the stratum corneum (Munro microabscesses) and/or the epidermis (spongiform pustules of Kogoj), when present, further supported the diagnosis of psoriasis, whereas spongiosis was generally minimal or absent in classic lesions. In contrast, the psoriasiform dermatitis cases showed a less stereotyped pattern, with more irregular epidermal hyperplasia, greater preservation or patchy alteration of the granular layer, less pronounced suprapapillary thinning, and more variable dermal vascular changes. Spongiosis was more prominent in eczematous lesions, while neutrophilic aggregates characteristic of classical psoriasis were generally absent or inconspicuous. Final classification was based on integration of these histopathological findings with the clinical presentation and the diagnosis of the underlying inflammatory dermatosis.

Clinical data retrieved for each case included disease duration, duration of the biopsied lesion, clinical severity, previous topical or systemic treatment, biologic exposure, and the treatment-free interval before biopsy. In the psoriasis group, disease duration ranged from 6 months to 30 years, while the duration of the biopsied lesions ranged from 2 months to 5 years. None of the patients had received biologic therapy. Previous treatment was predominantly topical; one patient had previously received methotrexate, which had been discontinued four years before biopsy. All psoriasis patients had a treatment-free interval of 3–4 weeks before biopsy (Table 2). In the psoriasiform dermatitis comparator group, disease duration ranged from 2 to 30 years and lesion duration from 6 months to more than 10 years. Previous treatments were topical, and all patients had a treatment-free interval of 3–4 weeks before biopsy (Table 3).

Clinical severity was recorded as documented in the medical records and categorized as mild, moderate, or severe. Where available, the corresponding documented clinical assessment was also recorded, including ESIF, PASI, and DLQI in individual psoriasis cases.

### 2.3. Primary Antibodies

Immunohistochemical staining was performed using commercially available primary antibodies directed against intercellular adhesion molecule-1 (ICAM-1/CD54), vascular cell adhesion molecule-1 (VCAM-1/CD106), tumor necrosis factor-alpha (TNF-α), interleukin-1 beta (IL-1β), interleukin-17A (IL-17A), Collagen IV, and S100B.

The antibodies used in this study are presented in Table 4.

Negative controls were processed using the same immunohistochemical protocol with omission of the primary antibody. Dedicated positive-control tissue sections were not used.

### 2.4. Tissue Processing and Immunohistochemistry

Archived biopsy specimens had been routinely fixed in 10% neutral-buffered formalin, processed, and embedded in paraffin. Serial sections (4 μm thick) were mounted on adhesive-coated glass slides.

For histopathological evaluation, sections were stained with hematoxylin and eosin (H&E) using standard laboratory protocols.

For immunohistochemical analysis, tissue sections were deparaffinized in xylene and rehydrated through graded ethanol solutions to water. Heat-induced epitope retrieval was performed in 10 mM citrate buffer (pH 6.0) using microwave heating at 95 °C for 10 min, followed by cooling at room temperature for 20 min. After washing in phosphate-buffered saline (PBS), endogenous peroxidase activity was blocked with 3% hydrogen peroxide, followed by incubation with 5% normal serum for 60 min at room temperature to minimize nonspecific antibody binding.

Slides were incubated overnight at 4 °C with the respective primary antibodies in a humidified chamber. Following PBS washes and incubation with the corresponding secondary antibodies, immunoreactivity was detected using the Novolink Polymer Detection System (RE7140K, Leica Biosystems, Nussloch, Germany). Visualization was achieved with 3,3′-diaminobenzidine (DAB) as chromogen, followed by hematoxylin counterstaining. Sections were then dehydrated, cleared, coverslipped, and examined using a Leica DM3000 light microscope. Images were acquired and analyzed using Leica Application Suite (LAS), version 4.13.

### 2.5. Immunohistochemical Evaluation

Immunohistochemical staining was independently evaluated by two experienced observers who were blinded to the clinical diagnosis. Whenever discrepant interpretations occurred, the slides were reviewed jointly until consensus was achieved. Formal interobserver agreement statistics (e.g., Cohen’s κ or intraclass correlation coefficient) were not calculated.

Staining intensity was assessed using a semi-quantitative four-point scoring system:

- 0 = negative staining;
- 1 = weak staining;
- 2 = moderate staining;
- 3 = strong staining.

Each marker was evaluated separately in four tissue compartments:

- membranous expression;
- cytoplasmic expression;
- nuclear expression;
- inflammatory infiltrate.

Membranous expression was defined as staining localized predominantly along the cell membrane, cytoplasmic expression as staining within the cytoplasm, and nuclear expression as staining localized within the nuclei. Inflammatory-infiltrate expression was defined as positive staining in inflammatory cells within the dermal and/or epidermal inflammatory infiltrate. Nuclear staining was recorded descriptively when present and was not interpreted as evidence of transcriptional activity or pathway activation.

A total immunohistochemical score (range, 0–12) was obtained for each marker by summing the scores from the four compartments. This composite score was used as a semi-quantitative analytical measure for between-group comparisons and ROC analyses and should not be interpreted as a previously validated diagnostic scoring system.

### 2.6. Statistical Analysis

Statistical analysis was performed using IBM SPSS Statistics version 31.0 (IBM Corp., Armonk, NY, USA).

Because immunohistochemical scores represented ordinal variables, data were expressed as medians and interquartile ranges (IQR). Between-group comparisons were performed using the Mann–Whitney U test. Effect sizes for the Mann–Whitney U tests were expressed as r and interpreted according to Cohen’s conventional thresholds.

Receiver operating characteristic (ROC) curve analysis was performed to evaluate the diagnostic performance of each biomarker. The area under the curve (AUC), 95% confidence intervals (95% CI), optimal cutoff values determined using Youden’s index, sensitivity, specificity, positive predictive value (PPV), and negative predictive value (NPV) were calculated. Confidence intervals were estimated using 1000 bootstrap resamples.

A two-sided *p*-value < 0.05 was considered statistically significant. Given the exploratory nature and small sample size of the study, no formal adjustment for multiple comparisons was applied; therefore, *p*-values should be interpreted as exploratory, with consideration of the potential for type I error.

### 2.7. Ethical Considerations

The study was conducted in accordance with the ethical principles of the Declaration of Helsinki. Archived formalin-fixed, paraffin-embedded tissue specimens obtained from the Department of Pathology, University Clinical Railways Hospital, Iași, Romania, were analyzed in an anonymized manner. Written informed consent for diagnostic procedures and the use of biological material for scientific purposes had been obtained from all patients at the time of biopsy. The study was approved by the Ethics Committee of University Clinical Railways Hospital, Iași, Romania (Approval no. 12449, 14 July 2026).

## 3. Results

### 3.1. Patient Characteristics

**Figure 1:**
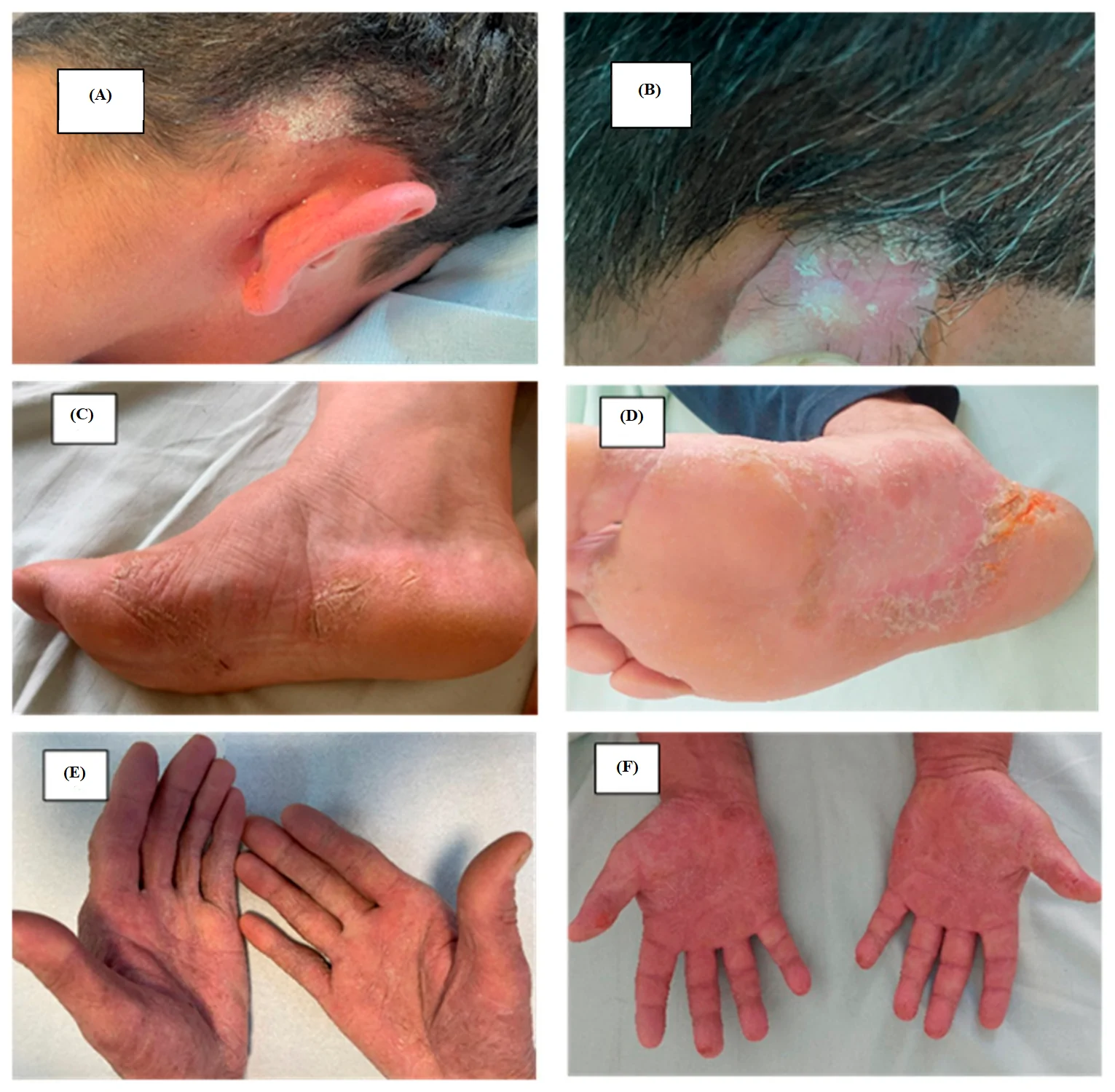
Representative clinical features of conditions included in the psoriasiform dermatitis comparator group (**A**,**C**,**E**) and psoriasis (**B**,**D**,**F**), involving the scalp, soles, and palms.

The study included 22 patients, evenly divided between psoriasis (*n* = 11) and the psoriasiform dermatitis comparator group (*n* = 11). The demographic and clinical characteristics of the cohort are summarized in Table 5. The groups were comparable with respect to age and sex. Representative clinical features of psoriasis and selected conditions within the psoriasiform dermatitis comparator group involving the scalp, palms, and soles are illustrated in Figure 1.

### 3.2. Total Immunohistochemical (IHC) Expression Scores

**Figure 2:**
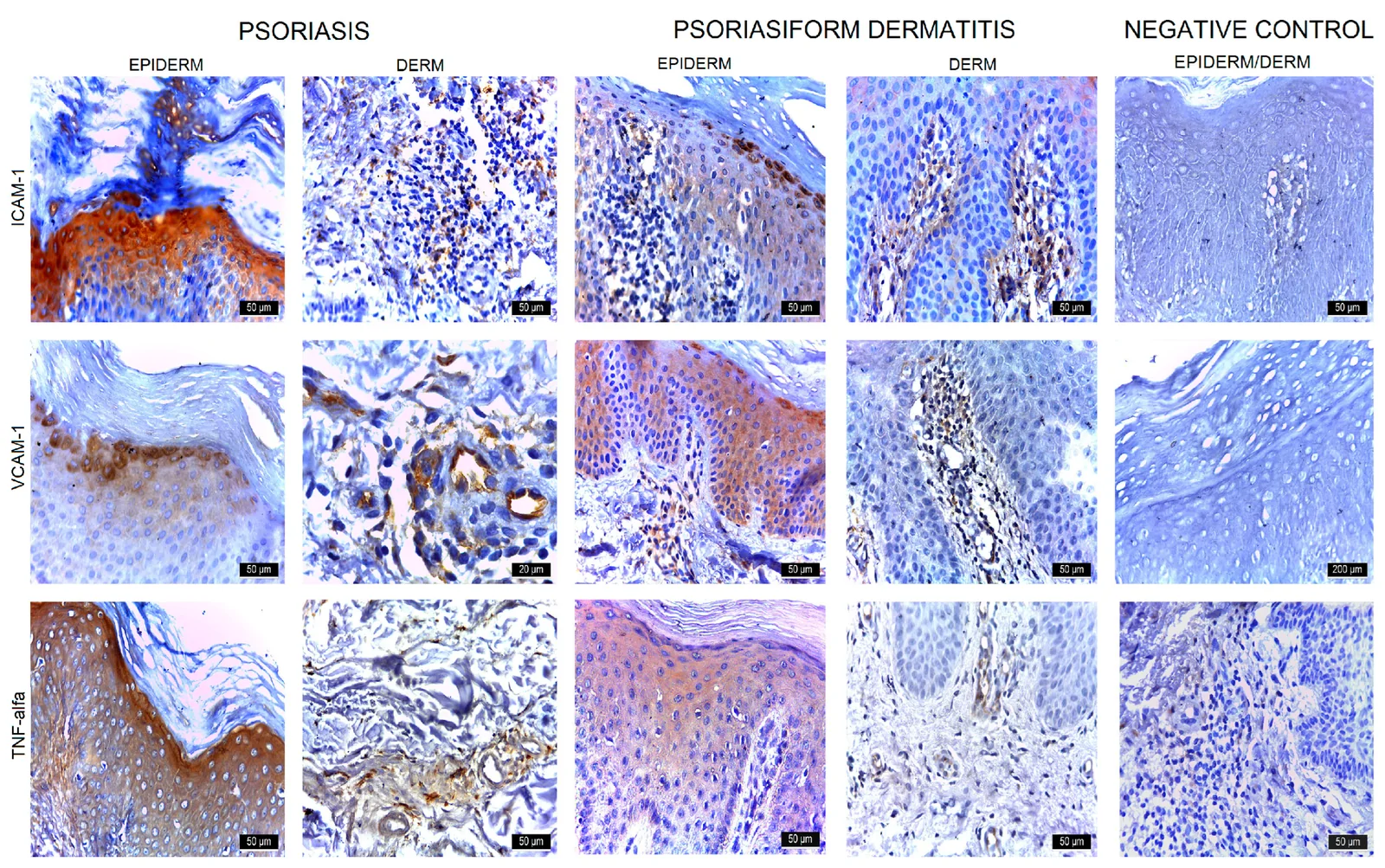
Representative immunohistochemical expression of ICAM-1, VCAM-1, and TNF-α in psoriasis vulgaris and the psoriasiform dermatitis comparator group. Representative photomicrographs show the immunohistochemical expression of ICAM-1, VCAM-1, and TNF-α in the epidermis and dermis. The right column shows the corresponding negative control sections processed without the primary antibody. Positive immunoreactivity is visualized as brown DAB staining with hematoxylin counterstaining. Scale bars are indicated in each panel.

**Figure 3:**
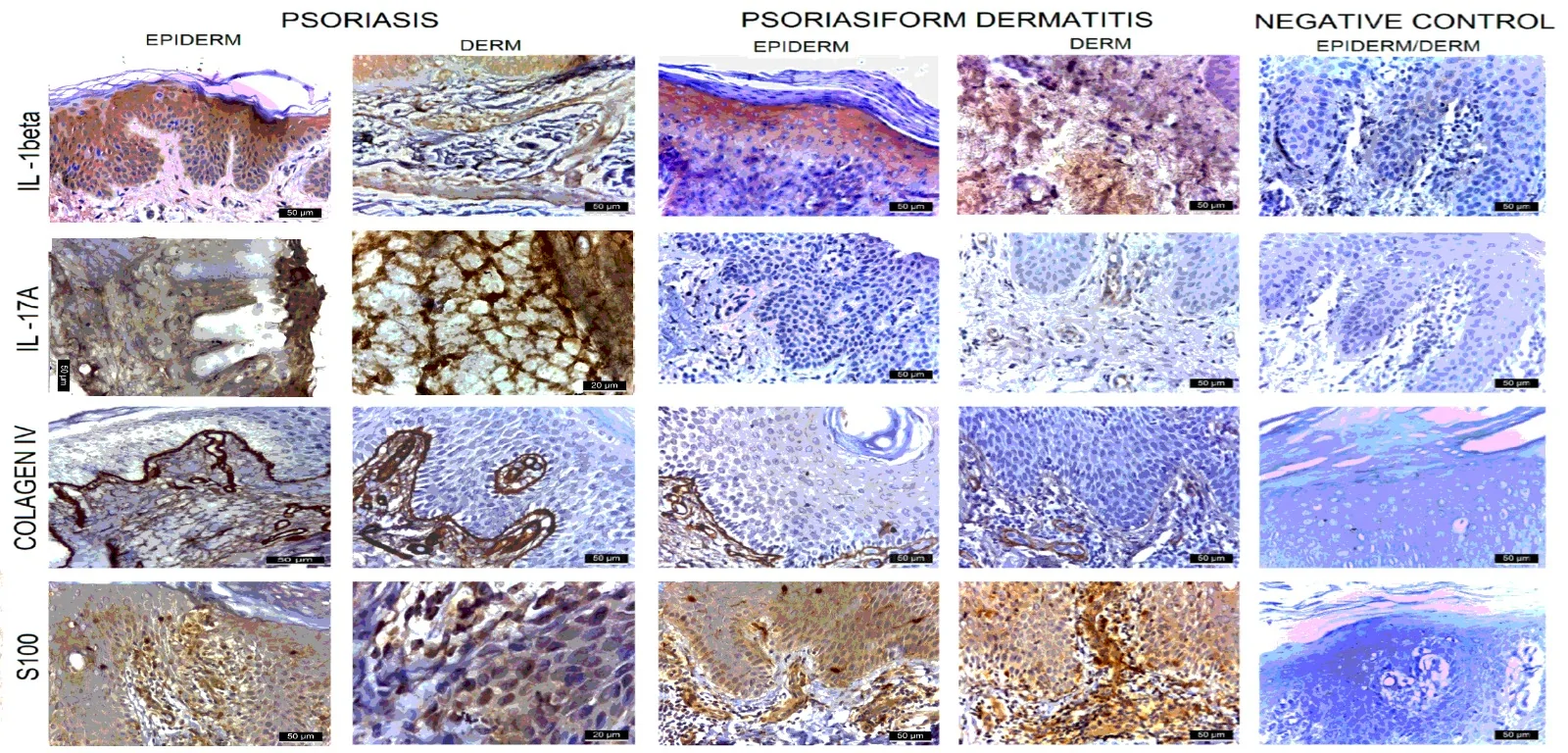
Representative immunohistochemical expression of IL-1β, IL-17A, Collagen IV, and S100B in psoriasis and the psoriasiform dermatitis comparator group. Representative photomicrographs illustrate the immunohistochemical expression of IL-1β, IL-17A, Collagen IV, and S100B in the epidermis and dermis. The right column shows the corresponding negative control sections processed without the primary antibody. Positive immunoreactivity is visualized as brown DAB staining with hematoxylin counterstaining. Scale bars are indicated in each panel.

The comparative analysis of total IHC scores (summed across all four tissue compartments) revealed significant differences in the expression profiles of inflammatory cytokines and adhesion molecules between the two groups. The results are summarized in Table 6, while representative immunohistochemical staining patterns are illustrated in Figure 2 and Figure 3.

Psoriasis lesions showed higher total expression scores for the main inflammatory cytokines. IL-17A and IL-1β demonstrated the largest effect sizes (*r* = 0.847), followed by TNF-α (*r* = 0.819). Among the adhesion molecules, ICAM-1 expression was significantly higher in psoriasis than in the psoriasiform dermatitis comparator group (*p* = 0.003, *r* = 0.637). VCAM-1 did not show a statistically significant difference between the two groups (*p* = 0.134). The structural basement membrane marker Collagen IV and the cellular marker S100B also showed no statistically significant differences in total expression between groups. Representative immunohistochemical staining patterns are shown in Figure 2 and Figure 3.

### 3.3. Compartment-Specific Expression Analysis

To further characterize the spatial distribution of immunohistochemical expression, each marker was evaluated separately within the membranous, cytoplasmic, nuclear, and inflammatory infiltrate compartments. The results of the compartment-specific analysis are summarized in Table 7, while representative immunohistochemical staining patterns are illustrated in Figure 2 and Figure 3.

**Figure 4:**
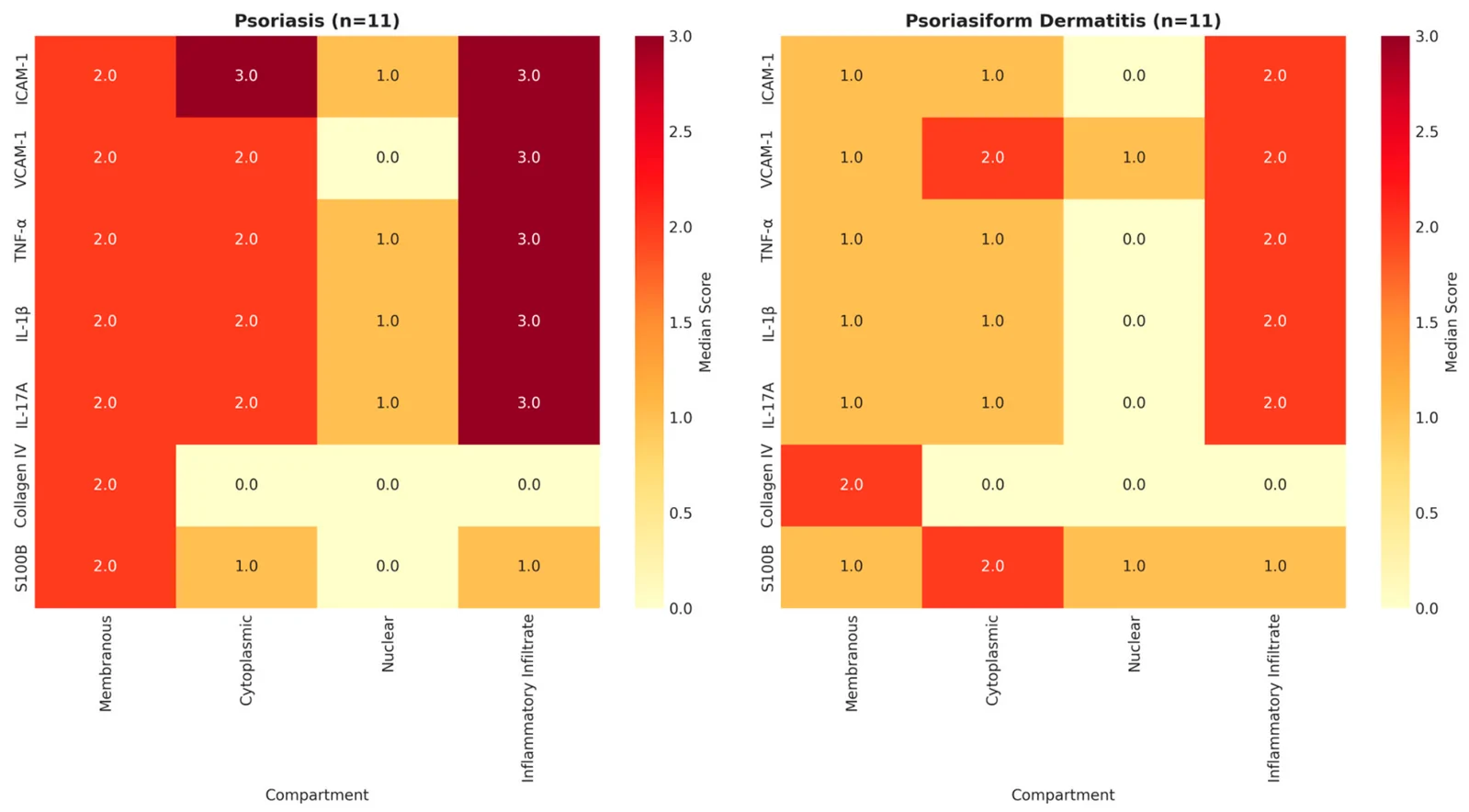
Compartment-Specific IHC Expression Heatmap.

Analysis of adhesion molecules showed that ICAM-1 expression was significantly higher in the membranous (*p* = 0.002), cytoplasmic (*p* = 0.016), and nuclear (*p* < 0.001) compartments in psoriasis. No statistically significant difference was observed in the inflammatory infiltrate compartment (*p* = 0.171). VCAM-1 expression was significantly higher in psoriasis in the membranous compartment (*p* = 0.003) and inflammatory infiltrate (*p* = 0.002), whereas the difference in cytoplasmic expression did not reach statistical significance (*p* = 0.065). Nuclear VCAM-1 staining was higher in the psoriasiform dermatitis comparator group (*p* = 0.047); this finding is reported descriptively and should not be interpreted as evidence of nuclear functional activity (Table 7, Figure 4).

Inflammatory cytokines IL-17A, TNF-α, and IL-1β showed significantly higher expression across all four evaluated compartments in psoriasis, including membranous, cytoplasmic, nuclear, and inflammatory infiltrate staining. These compartment-specific differences are summarized in Table 7 and Figure 4. Nuclear staining is reported descriptively and should not be interpreted as evidence of nuclear functional activity.

Collagen IV expression was restricted to the membranous compartment in both groups, with no significant differences detected (membranous *p* = 0.133; cytoplasmic, nuclear, and inflammatory infiltrate all *p* = 1.000). S100B demonstrated no significant differences in the membranous (*p* = 0.748), cytoplasmic (*p* = 0.401), or inflammatory infiltrate (*p* = 0.076) compartments. However, nuclear S100B expression was significantly higher in psoriasiform dermatitis (median 1 vs. 0, *p* = 0.002). The biological significance of this isolated finding remains uncertain and should be interpreted cautiously until confirmed in larger studies (Table 7, Figure 4).

### 3.4. Diagnostic Performance and ROC Analysis

**Figure 5:**
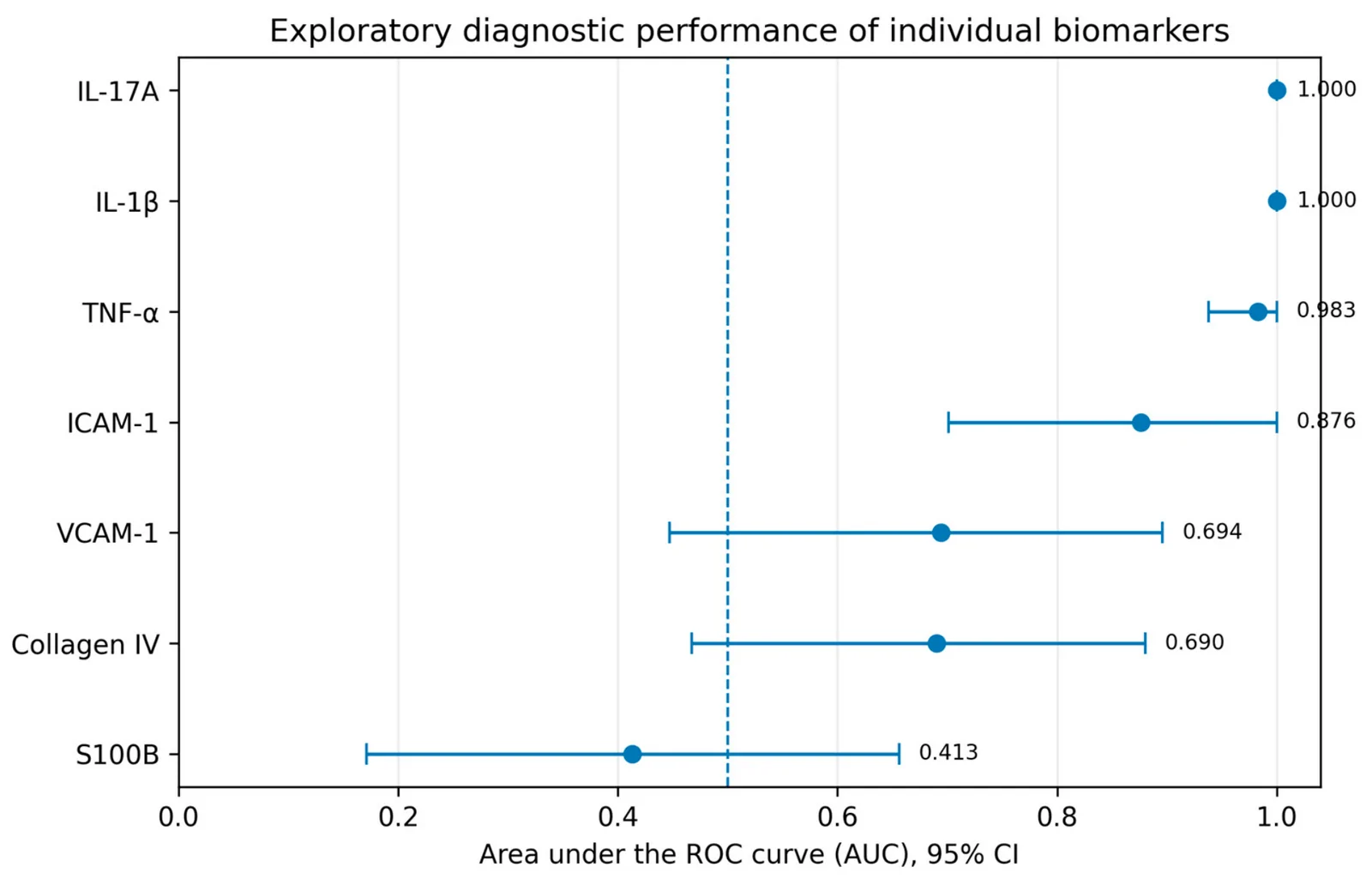
Exploratory diagnostic performance of individual immunohistochemical biomarkers for distinguishing psoriasis from the psoriasiform dermatitis comparator group. Points represent the area under the receiver operating characteristic curve (AUC), and horizontal error bars represent 95% confidence intervals. The dashed vertical line indicates an AUC of 0.5 (no-discrimination reference).

ROC curve analysis was performed to evaluate the diagnostic performance of each marker in differentiating psoriasis from the psoriasiform dermatitis comparator group (Figure 5). The inflammatory cytokines IL-17A and IL-1β each yielded an AUC of 1.000 in this cohort, with 100% sensitivity and specificity at their respective optimal cutoffs. Given the small sample size, these findings should be interpreted as exploratory. TNF-α also showed excellent discriminative ability (AUC = 0.983). Among the adhesion molecules, ICAM-1 showed good diagnostic performance (AUC = 0.876), providing 100% sensitivity and 72.7% specificity at a cutoff score of ≥7. VCAM-1 exhibited moderate diagnostic utility (AUC = 0.694), characterized by high sensitivity (100%) but low specificity (45.5%) at a cutoff of ≥6. Collagen IV and S100B showed limited discriminative performance as standalone markers. The ROC-derived cutoff values represent exploratory statistical estimates within this cohort and should not be interpreted as validated clinical diagnostic thresholds.

A combined biomarker panel comprising IL-17A, TNF-α, and ICAM-1 achieved an AUC of 1.000 within this study cohort. Addition of VCAM-1 did not further improve diagnostic performance. Given the small sample size and the absence of an independent validation cohort, these findings should be considered exploratory and hypothesis-generating and require external validation before any potential clinical application.

## 4. Discussion

The accurate histopathological differentiation between psoriasis and psoriasiform inflammatory dermatoses remains a significant challenge in routine dermatopathology. This study provides a comprehensive comparative analysis of key inflammatory and structural markers, showing higher expression of IL-17A, TNF-α, IL-1β, and ICAM-1 in psoriasis compared with the heterogeneous psoriasiform dermatitis comparator group. These findings suggest that these markers may have potential diagnostic value in distinguishing psoriasis from histopathologically overlapping inflammatory dermatoses. This study simultaneously evaluates adhesion molecules, inflammatory cytokines, and structural markers within a unified exploratory diagnostic framework.

### 4.1. IL-17A Expression in Psoriasis and Psoriasiform Dermatitis

Our findings are consistent with the central role of the IL-23/Th17 axis in the pathogenesis of psoriasis. IL-17A exhibited a large overall effect size (*r* = 0.847) and an AUC of 1.000 in this exploratory cohort, with significantly higher staining scores across all evaluated compartments in psoriasis compared with the psoriasiform dermatitis comparator group. IL-17A acts directly on keratinocytes, driving epidermal hyperplasia, downregulating differentiation markers such as filaggrin and loricrin, and inducing the production of antimicrobial peptides (β-defensins, S100A7/A8/A9) and chemokines (CXCL1, CXCL8) that recruit neutrophils [18]. The higher cytoplasmic IL-17A staining observed in psoriasis is consistent with the established involvement of the IL-17 pathway in psoriatic inflammation. Nuclear IL-17A staining was higher in psoriasis (median 1 vs. 0, *p* < 0.001); however, this finding was recorded descriptively and should not be interpreted as evidence of transcriptional activity, autocrine signaling, or a specific nuclear function.

The lower IL-17A staining observed in the heterogeneous psoriasiform dermatitis comparator group should be interpreted in the context of its diagnostic composition, which included atopic eczema, seborrheic dermatitis, and palmoplantar eczema. Atopic dermatitis is primarily associated with Th2-polarized inflammation involving IL-4 and IL-13, although variable Th17 involvement has also been described [27]. Th17 polarization may be more pronounced in specific atopic dermatitis phenotypes and populations [5]. However, because the comparator group in the present study was heterogeneous and included only five cases of atopic eczema, the observed between-group differences should not be interpreted as a direct psoriasis-versus-atopic dermatitis comparison.

### 4.2. TNF-α: The Inflammatory Amplifier

The higher TNF-α expression observed in psoriasis is consistent with its established role in inflammatory amplification. TNF-α synergizes with IL-17A to induce a broad array of pro-inflammatory genes in keratinocytes, a phenomenon described as the synergistic “IL-17 signature” [28]. This synergistic interaction promotes the expression of inflammatory mediators, including chemokines, antimicrobial peptides, and matrix metalloproteinases. In our exploratory ROC analysis, TNF-α showed a high AUC (0.983) for distinguishing psoriasis from the psoriasiform dermatitis comparator group. The higher dermal TNF-α staining observed in psoriasis is also consistent with the broader involvement of TNF-α in psoriatic inflammation and its reported association with systemic inflammatory comorbidities [29].

The compartment-specific analysis revealed significantly higher TNF-α staining in psoriasis across all four evaluated compartments: membranous (*p* = 0.003), cytoplasmic (*p* < 0.001), nuclear (*p* < 0.001), and inflammatory infiltrate (*p* = 0.002). Nuclear TNF-α staining was recorded descriptively and should not be interpreted as direct evidence of NF-κB activation, transcriptional activity, or a specific nuclear function. TNF-α/NF-κB signaling nevertheless represents an established component of inflammatory signaling in psoriasis [30].

### 4.3. IL-1β: Innate Immune Activation

IL-1β showed a large overall effect size (*r* = 0.847) and an AUC of 1.000 in this exploratory cohort, with significantly higher staining scores across all four evaluated compartments in psoriasis compared with the psoriasiform dermatitis comparator group. The higher IL-1β expression observed in psoriasis is consistent with the established involvement of innate immune pathways, including inflammasome-related signaling, in psoriatic inflammation [31]. IL-1β also contributes to inflammatory pathways that support Th17 responses, including interactions with dendritic-cell and IL-23–related signaling.

The lower IL-1β staining observed in the heterogeneous psoriasiform dermatitis comparator group should be interpreted cautiously given the inclusion of atopic eczema, seborrheic dermatitis, and palmoplantar eczema. Accordingly, the observed difference should not be interpreted as evidence of a uniform Th2-driven mechanism across the comparator group. Rather, these findings suggest differences in IL-1β expression between psoriasis and the inflammatory dermatoses included in this cohort, which require confirmation in larger and diagnostically stratified populations.

### 4.4. ICAM-1: Epidermal Leukocyte Trafficking

The comparative spatial analysis of adhesion molecules revealed distinct patterns of ICAM-1 expression between the two groups. ICAM-1 (CD54) showed an AUC of 0.876 (95% CI: 0.701–1.000) in this exploratory cohort. ICAM-1 staining was significantly higher in psoriasis in the membranous, cytoplasmic, and nuclear compartments, whereas no statistically significant difference was observed in the inflammatory infiltrate compartment (*p* = 0.171). The higher epidermal ICAM-1 expression observed in psoriasis is consistent with its established role in leukocyte adhesion and trafficking and with the inflammatory effects of cytokines such as TNF-α and IFN-γ [22]. These mechanisms may contribute to leukocyte recruitment within psoriatic lesions, including the characteristic accumulation of inflammatory cells in the epidermis.

Our findings are consistent with those of Marinović Kulišić et al. [24], who reported differential patterns of ICAM-1 expression in inflammatory skin lesions, and with earlier work by Lee et al. [25] describing increased ICAM-1 expression in psoriatic epidermis. In the present cohort, the significant between-group differences observed in the membranous and cytoplasmic compartments, together with the absence of a significant difference in the inflammatory infiltrate, suggest that epidermal ICAM-1 staining may contribute to the observed distinction between psoriasis and the heterogeneous psoriasiform dermatitis comparator group. Nuclear ICAM-1 staining was recorded descriptively and should not be interpreted as evidence of transcriptional activity or a specific nuclear function.

### 4.5. VCAM-1: Endothelial Activation and Potential Systemic Relevance

VCAM-1 (CD106) showed moderate discriminative performance (AUC = 0.694) in this exploratory cohort. Although the total VCAM-1 score did not differ significantly between the two groups (*p* = 0.134), compartment-specific analysis showed significantly higher staining in psoriasis in the membranous (*p* = 0.003) and inflammatory infiltrate (*p* = 0.002) compartments. Cytoplasmic VCAM-1 staining was also higher in psoriasis, although the difference did not reach statistical significance (*p* = 0.065). VCAM-1 is primarily associated with activated vascular endothelium and contributes to leukocyte adhesion and recruitment through interactions with VLA-4 (α4β1 integrin) [23]. The higher VCAM-1 staining observed in selected compartments in psoriasis is therefore consistent with the prominent inflammatory and vascular component of psoriatic lesions.

Nuclear VCAM-1 staining was higher in the psoriasiform dermatitis comparator group (median 1 vs. 0, *p* = 0.047). Given the borderline statistical significance and the uncertain biological significance of nuclear staining for this marker, this finding should be considered descriptive and interpreted cautiously. It should not be regarded as evidence of alternative signaling pathways, post-translational processing, intracellular trafficking, or a specific nuclear function and requires further investigation using complementary molecular approaches.

Beyond its tissue expression, circulating soluble VCAM-1 has been investigated in relation to systemic endothelial dysfunction and cardiovascular risk in psoriasis. Machoń et al. [32] reported an association between sVCAM-1 and cardiovascular risk-related parameters in patients with psoriasis. Although our tissue-level findings cannot establish a direct relationship with circulating VCAM-1 or cardiovascular outcomes, they are consistent with the broader involvement of endothelial activation in psoriatic inflammation [32]. Further studies integrating tissue expression, circulating biomarkers, and cardiovascular assessment are required to determine the potential clinical significance of these observations.

### 4.6. Collagen IV and S100B: Structural and Cellular Markers

Collagen IV and S100B showed no statistically significant differences in total immunohistochemical scores between psoriasis and the psoriasiform dermatitis comparator group (*p* = 0.110 and *p* = 0.504, respectively). For Collagen IV, compartment-specific analysis also showed no significant differences between the two groups, including the membranous compartment (*p* = 0.133). These findings indicate that differences in Collagen IV immunoreactivity were not demonstrated by the present immunohistochemical analysis.

S100B showed no significant differences in the membranous (*p* = 0.748), cytoplasmic (*p* = 0.401), or inflammatory infiltrate (*p* = 0.076) compartments. Nuclear S100B staining was significantly higher in the psoriasiform dermatitis comparator group (median 1 vs. 0, *p* = 0.002). The biological significance of this isolated finding remains uncertain, and nuclear staining should be considered descriptive rather than evidence of a specific nuclear function. Despite this compartment-specific finding, the total S100B score showed limited standalone discriminative performance (AUC = 0.413) in this exploratory cohort. Further studies in larger and diagnostically stratified cohorts are required to clarify the potential significance of S100B expression patterns.

### 4.7. Comparison with Published Literature and Broader Pathogenetic Context

Our findings are broadly consistent with the existing literature on immunohistochemical profiling of inflammatory skin diseases. Griffiths [26] described prominent ICAM-1 expression in psoriatic epidermis, supporting the involvement of adhesion molecules in leukocyte trafficking in psoriasis. Subsequent studies, including that of Watabe et al. [22], further investigated the role of ICAM-1/LFA-1 interactions in T-cell adhesion. The present study adds to these observations by comparatively evaluating ICAM-1 together with inflammatory cytokines and other tissue markers in psoriasis and a heterogeneous psoriasiform dermatitis comparator group and by assessing their exploratory diagnostic performance using effect-size and ROC analyses.

These tissue-level findings can be considered within the broader molecular context of chronic inflammatory skin diseases. Psoriasis has well-established genetic associations involving HLA-C*06:02, the late cornified envelope region, and genes related to IL-23/Th17 signaling [33,34,35,36,37,38,39]. In contrast, atopic dermatitis, which represented one component of our comparator group, has been associated with epidermal differentiation complex abnormalities, particularly filaggrin (FLG) loss-of-function variants, as well as Th2-related pathways [40,41,42,43,44,45,46,47,48]. Both psoriasis and atopic dermatitis impose substantial epidemiological, socioeconomic, and quality-of-life burdens, although their patterns of onset, population distribution, and clinical course differ [49,50,51,52,53,54,55,56,57,58,59,60,61,62]. Differences in epidermal barrier biology have also been extensively described, with filaggrin deficiency, lipid abnormalities, and IL-4/IL-13-mediated effects contributing to barrier dysfunction in atopic dermatitis, whereas altered keratinocyte proliferation and IL-17/IL-22-related inflammation contribute to epidermal abnormalities in psoriasis [40,43,63,64,65,66,67,68,69,70]. These distinct but partially overlapping inflammatory pathways have provided the rationale for targeted therapeutic strategies directed at IL-17/IL-23 signaling in psoriasis and IL-4/IL-13 or JAK-dependent pathways in atopic dermatitis [71,72,73,74,75,76,77,78,79,80,81,82,83,84,85,86,87,88,89]. However, because the comparator group in the present study also included seborrheic dermatitis and palmoplantar eczema, these broader psoriasis-versus-atopic dermatitis differences should be regarded as pathogenetic context rather than as a direct representation of the entire comparator group.

The higher IL-17A staining observed in psoriasis is also consistent with transcriptomic studies demonstrating prominent IL-17-related molecular signatures in psoriasis and greater molecular heterogeneity in atopic dermatitis [90]. Nevertheless, our immunohistochemical findings should not be considered direct validation of transcriptomic data, as the methodologies assess different levels of biological organization. IL-17A yielded an AUC of 1.000 in the present cohort, but this result should be interpreted cautiously given the small sample size, heterogeneous comparator group, and absence of an independent validation cohort. Accordingly, the diagnostic performance observed here should be considered exploratory and requires confirmation in larger, diagnostically stratified external cohorts.

### 4.8. Diagnostic Implications and Clinical Utility

These findings may have potential implications for dermatopathological practice, particularly in cases in which conventional H&E morphology shows overlapping psoriasiform features. In the present exploratory cohort, IL-17A and IL-1β showed the highest individual diagnostic performance, each yielding an AUC of 1.000, followed by TNF-α (AUC = 0.983) and ICAM-1 (AUC = 0.876). VCAM-1 showed more limited discriminative performance (AUC = 0.694). A combined panel comprising IL-17A, TNF-α, and ICAM-1 also yielded an AUC of 1.000 within this same cohort. However, these results were derived and evaluated in a small dataset without an independent validation cohort and should therefore be considered exploratory and hypothesis-generating rather than evidence for a validated diagnostic algorithm.

If confirmed in larger, diagnostically stratified and independent cohorts, selected immunohistochemical markers could potentially complement conventional histopathological assessment in diagnostically challenging cases. Such an approach should be considered an adjunct to, rather than a replacement for, clinicopathological correlation and standard H&E evaluation. The observed differences in inflammatory marker expression may also be relevant to the broader context of targeted therapies directed at distinct immune pathways in psoriasis and other inflammatory dermatoses. However, the present study was not designed to predict therapeutic response or treatment selection. Paradoxical or phenotype-shifting inflammatory reactions have been reported during targeted biologic therapy, illustrating the complex interactions between immune pathways [7,8,91].

### 4.9. Future Directions

Future studies should prioritize validation of these findings in larger, multicenter and diagnostically stratified cohorts, including independent validation populations, to assess their reproducibility and generalizability. The integration of digital pathology and AI-assisted image analysis could enable more objective and quantitative assessment of immunohistochemical expression, potentially reducing the limitations inherent to semi-quantitative scoring and allowing more precise evaluation of biomarker performance [91]. Correlation of tissue immunohistochemical expression with circulating biomarkers, such as sICAM-1 and sVCAM-1, and with clinical severity measures could further clarify the potential clinical relevance of these markers beyond histopathological discrimination. Finally, complementary molecular approaches, including spatial transcriptomic analyses, may help characterize the cellular sources and spatial relationships of cytokine and adhesion-molecule expression within the inflammatory tissue microenvironment.

### 4.10. Limitations

This study has several limitations. First, the sample size was small (*n* = 22), which limits statistical power and may contribute to overestimation of diagnostic performance, particularly for markers yielding very high AUC values. Second, the retrospective, single-center design limits the generalizability of the findings. Third, the psoriasiform dermatitis comparator group was heterogeneous, comprising atopic eczema, seborrheic dermatitis, and palmoplantar eczema. Although this composition reflects the spectrum of inflammatory dermatoses that may show overlapping psoriasiform features, the small number of cases within each diagnostic subgroup precluded meaningful subgroup-specific analyses.

Fourth, immunohistochemical expression was assessed using a semi-quantitative scoring system. Although evaluation was performed independently by two experienced observers blinded to the clinical diagnosis and discrepancies were resolved by consensus, formal interobserver agreement statistics, such as Cohen’s kappa or intraclass correlation coefficients, were not calculated. The absence of digital quantitative image analysis represents an additional methodological limitation. Fifth, given the exploratory nature and small sample size of the study, no formal adjustment for multiple comparisons was applied; consequently, the reported *p*-values should be interpreted cautiously in view of the potential for type I error.

Sixth, molecular methods such as transcriptomic or other complementary analyses were not performed; therefore, the observed immunohistochemical staining patterns, particularly nuclear staining, should not be interpreted as evidence of transcriptional activity or specific intracellular signaling mechanisms. Clinical severity was available only as categorical information (mild, moderate, or severe), limiting more detailed correlations between biomarker expression and disease severity. Finally, the ROC-derived cutoffs and the combined IL-17A/TNF-α/ICAM-1 panel were derived and evaluated within the same small cohort, without an independent validation population. Their diagnostic performance may therefore be overestimated and should be considered exploratory and hypothesis-generating until confirmed in larger, multicenter, diagnostically stratified external cohorts.

## 5. Conclusions

In conclusion, this preliminary exploratory study identified differences in the immunohistochemical expression profiles of psoriasis and the heterogeneous psoriasiform dermatitis comparator group despite their overlapping histopathological features. IL-17A, IL-1β, TNF-α, and ICAM-1 showed the greatest between-group differences and the highest diagnostic performance within this cohort, whereas VCAM-1 may provide complementary diagnostic information. Collagen IV and S100B showed limited discriminative performance based on their total immunohistochemical scores. These findings suggest that selected inflammatory and adhesion-related markers may have potential as adjuncts to conventional histopathological assessment in diagnostically challenging cases. However, given the small sample size, heterogeneous comparator group, and absence of independent validation, the diagnostic performance and proposed biomarker combinations should be considered exploratory and hypothesis-generating. Validation in larger, multicenter, diagnostically stratified independent cohorts is required before any potential clinical implementation.

## Figures and Tables

**Table 1:** Study Groups.

Group	Diagnosis	Number of Cases
Study group	Psoriasis vulgaris	11
Comparator group	Atopic eczema	5
Comparator group	Seborrheic dermatitis	3
Comparator group	Palmoplantar eczema *	3

* One palmoplantar eczema case was specifically classified as hyperkeratotic palmoplantar eczema.

**Table 2:** Patient-Level Clinical Characteristics and Treatment History of the Psoriasis Group.

No.	Disease Duration	Lesion Duration	Severity	Previous Topical Treatment	Biologic Treatment	Treatment-Free Interval Before Biopsy
1	4 years	2 months	Moderate	Urea	None	4 weeks
2	4 years	2 months	Moderate	Diprogenta	None	4 weeks
3	1 year	1 year	Moderate	Advantan	None	4 weeks
4	2 years	6 months	Moderate	Elocom	None	4 weeks
5	30 years	7 months	Severe	Advantan	None	4 weeks
6	20 years	2 years	Mild	Advantan	None	4 weeks
7	2 years	>6 months	Mild	Mometasone (MTX discontinued 4 years previously)	None	4 weeks
8	22 years	5 years	Moderate	Diprosalic	None	4 weeks
9	10 years	>2 years	Moderate	Elosalic	None	4 weeks
10	6 months	6 months	Moderate	Mometasone	None	3 weeks
11	7 years	>1 year	Severe	Daivobet	None	3 weeks

**Table 3:** Patient-Level Clinical Characteristics and Treatment History of the Psoriasiform Dermatitis Comparator Group.

No.	Diagnosis/Factors	Disease Duration	Lesion Duration	Severity	Previous Topical Treatment	Treatment-Free Interval Before Biopsy
1	Hyperkeratotic palmoplantar eczema	4 years	4 years	Severe	Elocom, urea, salicylic acid	3 weeks
2	Atopic eczema	10 years	6 months	Severe	Advantan	3 weeks
3	Seborrheic dermatitis	5 years	>1 year	Severe	Diprosalic	3 weeks
4	Seborrheic dermatitis	11 years	>1 year	Severe	Xamiol	3 weeks
5	Atopic eczema	4 years	>6 months	Moderate	Dermovate	3 weeks
6	Seborrheic dermatitis	10 years	>6 months	Moderate	Dermovate	3 weeks
7	Palmoplantar eczema	3 years	>6 months	Moderate	Urea	3 weeks
8	Atopic eczema	2 years	>6 months	Moderate	Advantan	3 weeks
9	Atopic eczema	10 years	>6 months	Moderate	Advantan	4 weeks
10	Palmoplantar eczema	6 years	>1 year	Moderate	Elocom	4 weeks
11	Atopic eczema	30 years	>10 years	Severe	Advantan	4 weeks

**Table 4:** Antibodies used for immunohistochemical staining.

Marker	Clone/Catalog No.	Host	Manufacturer	Dilution
ICAM-1 (CD54)	G-5 (sc-8439)	Mouse monoclonal	Santa Cruz Biotech-nology, Inc. (Dallas, TX, USA)	1:200
VCAM-1 (CD106)	M/K-2 (sc-18864)	Rat monoclonal	Santa Cruz Biotech-nology, Inc. (Dallas, TX, USA)	1:500
TNF-α	AAR33	Rabbit polyclonal IgG	Bio-Rad Laboratories, Inc. (Hercules, CA, USA)	1:250
IL-1β	AAR15G	Rabbit	Bio-Rad Laboratories, Inc. (Hercules, CA, USA)	1:250
IL-17A	eBio17B7	Rat monoclonal	eBioscience/Thermo Fisher Scientific (Waltham, MA, USA)	1:100
Collagen IV	ab6586	Rabbit polyclonal	Abcam (Cambridge, UK)	1:400
S100B	C48942	Rabbit monoclonal	Signalway Antibody (College Park, MD, USA)	1:1000

**Table 5:** Patient Demographics and Clinical Characteristics.

Characteristic		Psoriasis (*n* = 11)	Psoriasiform Dermatitis Comparator Group (*n* = 11)	*p*-Value
**Age (years), median (range)**		45 (28–68)	42 (22–65)	0.654
**Sex, *n* (%)**	Male	6 (54.5%)	5 (45.5%)	0.680
	Female	5 (45.5%)	6 (54.5%)	
**Biopsy site, *n* (%)**	- Trunk	4 (36.4%)	3 (27.3%)	1.000
	- Upper extremities	4 (36.4%)	5 (45.5%)	
	- Lower extremities	3 (27.3%)	3 (27.3%)	

**Table 6:** Total IHC Scores: Psoriasis versus Psoriasiform Dermatitis Comparator Group.

Marker	Psoriasis Median (IQR)	Psoriasiform Dermatitis Median (IQR)	Mann–Whitney U	*p*-Value	Effect Size (*r*)	Significance
**IL-17A**	8 (8–9)	4 (3–5)	121.0	<0.001	0.847	***
**IL-1β**	8 (7–10)	4 (3–6)	121.0	<0.001	0.847	***
**TNF-α**	8 (7–10)	4 (3–6)	119.0	<0.001	0.819	***
**ICAM-1**	9 (7–9)	4 (3–7)	106.0	0.003	0.637	**
**VCAM-1**	7 (6–10)	6 (4–8)	83.5	0.134	0.322	ns
**Collagen IV**	2 (1–3)	2 (1–2)	83.5	0.110	0.322	ns
**S100B**	4 (3–6)	5 (4–6)	50.0	0.504	0.147	ns

Note: IQR = Interquartile Range; ns = not significant; ** *p* < 0.01; *** *p* < 0.001.

**Table 7:** Compartment-Specific IHC Expression Analysis in Psoriasis and the Psoriasiform Dermatitis Comparator Group.

Marker	Compartment	Psoriasis Median	Psoriasiform Dermatitis Comparator Group Median	*p*-Value	Significance
**ICAM-1**	Membranous	2	1	0.002	**
	Cytoplasmic	3	1	0.016	*
	Nuclear	1	0	<0.001	***
	Inflammatory Infiltrate	3	2	0.171	ns
**VCAM-1**	Membranous	2	1	0.003	**
	Cytoplasmic	2	2	0.065	ns
	Nuclear	0	1	0.047	*
	Inflammatory Infiltrate	3	2	0.002	**
**TNF-α**	Membranous	2	1	0.003	**
	Cytoplasmic	2	1	<0.001	***
	Nuclear	1	0	<0.001	***
	Inflammatory Infiltrate	3	2	0.002	**
**IL-1β**	Membranous	2	1	0.001	**
	Cytoplasmic	2	1	<0.001	***
	Nuclear	1	0	<0.001	***
	Inflammatory Infiltrate	3	2	0.001	**
**IL-17A**	Membranous	2	1	0.002	**
	Cytoplasmic	2	1	<0.001	***
	Nuclear	1	0	<0.001	***
	Inflammatory Infiltrate	3	2	<0.001	***
**Collagen IV**	Membranous	2	2	0.133	ns
	Cytoplasmic	0	0	1.000	ns
	Nuclear	0	0	1.000	ns
	Inflammatory Infiltrate	0	0	1.000	ns
**S100B**	Membranous	2	1	0.748	ns
	Cytoplasmic	1	2	0.401	ns
	Nuclear	0	1	0.002	**
	Inflammatory Infiltrate	1	1	0.076	ns

Note: ns = not significant; * *p* < 0.05; ** *p* < 0.01; *** *p* < 0.001. All *p*-values from Mann–Whitney U test.

## Data Availability

The original contributions presented in this study are included in the article. Further inquiries can be directed to the corresponding author.

## Abbreviations

The following abbreviations are used in this manuscript:

ICAM-1	Intercellular adhesion molecule-1
VCAM-1	Vascular cell adhesion molecule-1
TNF-α	Tumor necrosis factor-alpha
IL	Interleukin
Th	T helper
AD	Atopic dermatitis
H&E	Hematoxylin and eosin
LFA-1	Lymphocyte function-associated antigen-1
VLA-4	Very late antigen-4
FFPE	Formalin-fixed, paraffin-embedded
PBS	Phosphate-buffered saline
HRP	Horseradish peroxidase
DAB	3,3′-diaminobenzidine
IQR	Interquartile ranges
ROC	Receiver Operating Characteristic
AUC	Area under the curve
CI	Confidence intervals
PPV	Positive predictive value
NPV	Negative predictive value
IHC	Immunohistochemical
